# Notes and News

**Published:** 1898-03-05

**Authors:** 


### Notes and News.

(Collected and Communicated.)

HOSPITAL MEETINGS.

The Dental Hospital of London.—The Annual General Meeting of Governors will be held at the Hospital on Thursday, March 17th, at 5.30.
Royal Westminster Ophthalmic Hospital.—Annual General Meeting, Friday, March 4th, 4 p.m., at the Hospital.
National Hospital for Diseases of the Heart.—Annual General Meeting, at the Hospital, Thursday, March 3rd, at 4 p.m.
St. Mary's Hospital.—Annual Meeting, March 18th, 4.30 p.m., at the Hospital.
West London Hospital.—Annual Meeting, Wednesday, March 16th, at 4 p.m.
Royal Eye Hospital, Southwark.—Annual General Meeting, Wednesday, March 23rd, 4 p.m.
St. Peter's Hospital, Covent Garden.—Annual Meeting, Thursday, March 3rd, at 5 p.m., at the Hospital.

Mr. C. W. Lea, among numerous legacies to Worcester charities, has left £10,000 to the infirmary.

A dance in aid of Charing Cross Hospital Convalescent Home will be held at the Empress Rooms, Royal Palace Hotel, Kensington, on April 27th.

At a general meeting of the Marwari community at Howrah, near Salkia, Babu Hurdutroy Chamria, honorary magistrate of Howrah, presented a site for the erection of a Marwari Caste Plague Hospital.

The Victoria Ward of the Foreign Hospital at Nice, built to commemorate the Jubilee, is now complete, the furniture having been given by an anonymous lady, and it is hoped the building will be opened by a member of the Royal Family during the Queen's stay at Nice.

A festival ball in aid of the Italian Hospital, Queen Square (an entirely new building now in course of construction), will be held on May 2nd at the Galleries of the Royal Institute of Painters in Water Colours, Piccadilly, W.

According to the Army Medical Report for 1896, which has just been issued, venereal disease continued to cause a large amount of sickness in the British Army, especially in India. Although the ratios were lower than in 1895 they were distinctly above the average for the ten preceding years. Out of the European garrison of about 70,000 men, no less than 3,162 were constantly non-effective in consequence of venereal disease, while 448 were invalided for secondary syphilis during the year.

The founder of the Poplar Hospital, Mr. Charles Mare, once a wealthy, and always a philanthropic and generous, man, has just died under such destitute circumstances as rendered a public subscription necessary to save him from a pauper funeral. His wife, once a famous Court beauty and Maid of Honour to the Queen, shared his poverty. It was owing to the friendly offices of Nurse Coburn-Coulton and Dr. Stonham that the sum necessary to give him a respectable funeral was collected.

The *Practitioner* says that the late Dr. Pean, in his surgical practice, took for his motto Danton's "*De l'audace, de l'audace, surtout de l'audace.*" Hence many brilliant successes which were trumpeted abroad, and not a few dismal failures, of which less was heard. His works were, it is said, mostly written by his pupils, and throughout his career he kept steadily in view the object with which he set out—to put money in his purse. It is also said that he was the original of "Dr. Tant pour Cent" in Gyp's "Les Chers Docteurs," all of which is rather rough on poor Pean, who, coming as he did from peasant stock, no doubt had the love of money characteristic of the French peasant. But where would France be now but for these thrifty and acquisitive instincts which are so engrained in its peasantry as to tinge their lives even when they become wealthy and successful.

A series of cases of milk poisoning attributed to the use of Pasteurised milk are recorded in the *New York Medical Record*, and it is pointed out how large is the number and variety of bacteria which are not destroyed by this process. Even the acid-producing bacteria are left in sufficient profusion to proliferate rapidly unless the Pasteurised milk is kept on ice, a thing which it is obviously impossible to ensure. In milk, however, which had been sterilised at a temperature of 90 deg. or 92 deg. C., the acidity remained stationary for from three days to three weeks, without ice, in the warmest weather. The conclusion arrived at by the author of the paper is that Pasteurised milk is an uncertain, and in some cases a very dangerous, food for infants, and that in the domestic sterilisation of milk it should be heated at least to a temperature of 90 to 100 deg. C., the latter being the preferable temperature, for ten minutes.

At an inquest recently held at Mile End a condition of affairs in connection with medical aid associations came out which is clearly illegal. The mother of the deceased gave evidence to the effect that she called the doctor whose name appeared on the card, and that he attended the child until its death. He, however, said he could not give a death certificate, and for this referred her to a medical man who in turn referred her to the coroner. At the inquest it was stated that this "doctor" was an M.D. of Vienna, but was not a registered medical practitioner. The coroner made some strong remarks about such a person being employed in such a capacity. He said, "I think it most improper for the Medical Aid Company to employ a doctor who cannot give a death certificate. It misleads poor people who pay for the attendance of a doctor." We think that in a case of this sort the coroner would have done well to draw the attention of the Medical Council to the matter, for the Medical Act is definite, at least, on this—that no unregistered person may hold any appointment as physician, surgeon, or other medical officer, to any friendly or other society for affording mutual relief in sickness, infirmity, or old age.

